# Not Quite Right: Representations of Eastern Europeans in ECJ Discourse

**DOI:** 10.1007/s10767-020-09368-2

**Published:** 2020-06-10

**Authors:** Dagmar Rita Myslinska

**Affiliations:** Goldsmiths, University of London, New Cross, London, SE14 6NM UK

**Keywords:** Critical discourse analysis, ECJ jurisprudence, Legal discourse, Free movement, Postcolonialism, Central and Eastern Europe, EU

## Abstract

Although the increasing responsiveness of the Court of Justice of the European Union (the ‘ECJ’) jurisprudence to western Member States’ concerns regarding Central and Eastern European (‘CEE’) nationals’ mobility has garnered academic attention, ECJ *discourse* has not been scrutinised for how it approaches the CEE region or CEE movers. Applying postcolonial theory, this article seeks to fill this gap and to explore whether there are any indications that ECJ discourse is in line with the historical western-centric inferiorisation of the CEE region. A critical discourse analysis of a set of ECJ judgments and corresponding Advocate General opinions pertaining to CEE nationals illustrates not only how the ECJ adopts numerous discursive strategies to maintain its authority, but also how it tends to prioritise values of the western Member States, while overlooking interests of CEE movers. Its one-sided approach is further reinforced by referring to irrelevant facts and negative assumptions to create an image of CEE nationals as socially and economically inferior to westerners, as not belonging to the proper EU polity and as not quite deserving of EU law’s protections. By silencing CEE nationals’ voices, while disregarding the background of east/west socio-economic and political power differentials and precariousness experienced by many CEE workers in the west, such racialising discourse normalises ethnicity- and class-based stereotypes. These findings also help to contextualise both EU and western policies targeting CEE movers and evidence of their unequal outcomes in the west, and are in line with today’s nuanced expressions of racisms. By illustrating the ECJ’s role in addressing values pertinent to mobile CEE individuals, this study facilitates a fuller appreciation of the ECJ’s power in shaping and reflecting western-centric EU identity and policies. Engaging with such issues will not only allow us to better appreciate—and question—the ECJ’s legitimacy, but might also facilitate a better understanding of power dynamics within the EU. This study also makes significant theoretical and methodological contributions. It expands (and complicates) the application of postcolonial theory to contemporary intra-EU processes, while illustrating the usefulness of applying critical discourse analysis to exploring differentiation, exclusion, subordination and power within legal language.

## Introduction

Oppressive language does more than represent violence; it is violence; does more than represent the limits of knowledge; it limits knowledge. Whether it is obscuring state language or the faux-language of mindless media; whether it is the proud but calcified language of the academy … the malign language of law-without-ethics, or language designed for the estrangement of minorities, … it must be rejected, altered and exposed (Morrison 1994, p. 16).

[L]egal language is never objective. If reality is indeed created through language, one can say that this is even more so in the field of law since law, in essence, is language (Kalimo et al. 2018, p. 288).

Brexit supporters frequently portrayed Central and Eastern European (‘CEE’)^1^ movers as criminals and murderers (e.g. Vote Leave 2016; Leave.EU 2016) accustomed to ‘living like animals’ (Bienkov 2013). Mainstream British political discourse has also been filled with anti-CEE remarks (e.g. Straw 2013; Cameron 2013; Allen 2016), reminiscent of UK politics a century ago against ‘aliens from Eastern Europe’ whose ‘habits had a demoralising effect’ on Britons (Cohen 2006, p. 71). This recent rhetoric has intersected with negative British media portrayals of CEE movers as foreigners and purveyors of crime and disease (Fox et al. 2012; Samaluk 2016; Spigelman 2013; Drzewiecka et al. 2014).

Anti-CEE discourse has not been limited, however, to the Eurosceptic UK. CEE movers have also faced negative public climate in other western EU Member States (Commission 2011). It has become acceptable for mainstream western politicians and the media to refer to CEE mobility as ‘benefit tourism’ and ‘poverty immigration’ (Galgóczi et al. 2011; Poptcheva 2014) of ‘foreigners’ (Kostakopoulou 2014), ‘criminals’ and ‘drunks’ (e.g. Financial Times 2011), and to denigrate CEE States as backwards and parasitic (Sobis 2016; Bell 2015). In a 2013 letter to the European Council for Justice and Home Affairs, representatives of Austria, Germany, the Netherlands and the UK complained about the alleged financial abuse of western ‘benefit magnet’ Member States by CEE ‘immigrants’ (Miki-Leitner et al. 2013)—a term that, under EU law, applies only to those from outside the EU. This backlash has relied on ‘regimes of representation that portray’ CEE movers ‘as the bearers of alien customs and practices’, suited for ‘permanent exclusion … from … the imagined national communit[ies]’ (Virdee and McGeever 2018, p. 1808).

Despite frequent rhetoric about equality, fundamental rights and unity (Smismans 2010; Myslinska 2019a), EU bureaucrats have also been disseminating western-centric discourse. For example, this has been observed in EU cultural policies (Sassatelli 2009), and in official statements made before (Böröcz and Kovacs 2001) and on the eve of the Eastern Enlargement (Behr 2007; Engel-Di Mauro 2006). In such discourse, CEE States have been represented as irrational and in need of westernisation, and their accession to the EU was framed as a civilising mission. Through the Eastern Enlargement process, western EU States were ‘opening up to new people’ (Commission 2004) and preparing to ‘embrace widely different … cultures’ (Kok 2003, p. 68). The CEE region had to be ‘rescued’ by western Europe, which was to serve as a ‘mentor’ and ‘beacon to guide the applicants’ (Commission 2003) in their efforts to partake of some of the achievements of western Europe. Only through a constant process of improvement could CEE States hope to overcome not only economic, but also ‘social disparities’ between them and the west (Europe Agreement,^2^ Preamble).

Moreover, EU institutions have been tolerating negative stereotyping, outcome inequalities and racism faced by CEE movers in EU-15^3^ States. For example, Members of the European Parliament have blamed CEE States for ‘exporting their crime’ (Parliament 2013b), especially prostitution (Parliament 2013a, p. 61), and for spreading diseases (such as tuberculosis) to the west (Parliament 2014, p. 184). When responding to such comments, EU institutions have not sought to dispel their racist stereotypes. The EU has also been failing to condemn curbs on CEE movers’ rights in EU-15 States as violations of their fundamental rights (e.g. Parliament 2014b, 2014c).

EU institutions have also given little attention to reports^4^ brought to their attention of CEE movers’ experiences of inequalities and discrimination (e.g. EUMC 2006; ENAR 2009; Parliament 2014a; see generally Currie 2007). Even EU reports devoted specifically to combating inequalities and protecting fundamental rights have continued to reinforce EU discourse that overlooks unequal experiences of CEE movers (e.g. Commission 2008, 2009, 2014a, 2016, 2017, 2018, 2018a, 2019; Parliament 2005; FRA 2012, 2013, 2014, 2018). Moreover, when acknowledging—on rare occasions—such experiences suffered by CEE movers, EU institutions have not called for policy measures to protect them. For example, despite concluding that most victims of prejudice and discrimination in EU-15 States in 2011–2012 have been CEE movers, the Commission failed to acknowledge a need for greater EU and national policy efforts to protect them (Commission 2013; see also Commission 2013a, pp. 98–99). Notably, a recent report devoted specifically to EU citizen’s rights noted that EU movers experience discrimination in accessing employment, housing, banking services and education (FRA 2018, p. 50), but did not mention the need for their greater equality protections.

Of course, CEE movers have also been negatively impacted by direct EU *policies*. Both 2004 and 2007 accession treaties allowed Member States to impose transitional mobility derogations on CEE workers for up to 7 years after accession. During the last decade, moreover, all EU institutions have increasingly adopted policies to reduce the rights of economically inactive movers and jobseekers, and have tolerated Member States’ curbs of rights afforded to all categories of movers (Myslinska 2019). Since post-04 mobility has been largely east to west (Ritzen et al. 2017), all such initiatives would have had a disproportionate effect on CEE nationals (Myslinska 2019).^5^ Moreover, the negative impact of the jurisprudence of the Court of Justice of the European Union (‘ECJ’) on CEE movers’ rights has garnered increasing academic attention (e.g. Currie 2009; Dougan 2013; O’Brien 2015; Myslinska 2019; Zahn 2015). Notably, the ECJ has become more responsive to EU-15 States’ arguments that they have served as magnets for CEE movers due to their (allegedly) generous welfare policies, despite the lack of data to support such claims.^6^

All such rhetoric and policies are in line with the long-standing mainstream scholarly and popular constructions of European history (Gatrell 2019) and of the concept of ‘Europe’ (Hansen 2002), which have been replete with popular anti-Slavic sentiment. In such representations, the west is portrayed as a superior site of civilisation, wealth, peace and modernity (Bhambra 2019; Burgess 2002; Sassatelli 2002), and the east as primitive (Korek 2007; Todorova 2003; Verdery 2002) peripheries of ‘Europe’, filled with inhabitants ‘fit only for slavery’ (Wolff 1994: 10ff).^7^ This view has been propagated throughout the Middle Ages (Delanty 2003) and the Enlightenment (Wolff 1994), through the concept of Mitteleuropa a century ago (Meyer 1955), during the Cold War (Todorova 2003), and as part of the process leading to the EU’s Eastern Enlargement in 2004 and 2007 (Böröcz and Kovacs 2001). Similarly, European lawyers and legal academics have traditionally tended to privilege the concerns of north-western EU States (Kukovec 2015).

Unlike the substantive impact of ECJ rulings, the Court’s *discourse* has not been scrutinised^8^ for how it approaches the CEE region or CEE movers. This is likely in part due to the fact that the ECJ’s role is commonly understood as to uphold the checks and balances built into the EU constitutional legal order, without intruding into the political sphere (Lenaerts 2013).^9^ It is true that ECJ judgements, akin to the civil law tradition, tend to focus on detailed statutory analyses with little background or contextual discussion and little *obiter dicta*,^10^ and the language used by the ECJ tends to be formal and sparse (Kalimo et al. 2018), likely reflecting the fact that rulings are reached through a consensus of judicial panels^11^ that speak with one voice, without issuing concurring or dissenting opinions. However, studies of ECJ discourse have revealed some fruitful findings that shed light on the Court’s legitimacy and on what values it propagates (e.g. id; Dabbagh 2017). More generally, by exposing unformulated legal bases and hidden assumptions behind judicial decisions, close scrutiny of *dicta* supplements and contextualises doctrinal analysis (e.g. Zalesne 2013). Such insights are especially helpful to understanding how case-law reflects cultural assumptions and how it is used to maintain social power and dominant ideologies.

I seek to explore how ECJ discourse has been conceptualising the CEE region and its nationals,^12^ and whether there are any indications that it is in line with the historical and contemporary western-centric rhetoric discussed above. More generally, my goal is to try to situate the ECJ’s role in addressing values and interests pertinent to mobile CEE individuals within the context of today’s intra-EU socio-economic and political developments. Engaging with such topics will not only allow us to better appreciate the ECJ’s legitimacy and its role in shaping and reflecting EU identity and policies, but might also facilitate a better understanding of today’s nuanced expressions of racisms within Europe and of any shortcomings of the EU project’s promises of equality. As explained below, I rely on critical discourse analysis and postcolonial theory in this study because they are particularly well-suited to explorations of how dominant values, ideologies, identities and positions of power get re-produced.

## Theoretical Framework

Postcolonial theorists have exposed how western discourse has propagated an Orientalist gaze of the east as inferior to the west (Said 1995). Facilitated by inculcating a servile mentality on colonial subjects (Fanon 1988), this process has produced lasting negative political, economic and social effects on postcolonial environments and on westerners’ view of their former subjects (e.g. Anghie 2007; Chimni 2013; Chakrabarty 2000; Kerner 2018). Although much academic attention has been devoted to the Global North’s and West’s imperialism of the Global South and the far East, historical intra-European relations have also been subjected to postcolonial critique—such as English colonialism of the Celts and German hegemony over Central Europe (Mahmud 2007).

*Post*-colonialism does not imply that colonialism is over. Rather, it looks at the continuing effects of historical practices and constructs on present racial stratifications, political dominations and systems of knowledge and power (Go 2018), and seeks to disrupt them by recognising non-western histories, interests and voices (Chakrabarty 2000; Spivak 1999). Europe’s idea of itself still depends on (western) imperialist attitudes (Balibar 1991; Fitzpatrick 2001). Notably, postcolonial theorists question western-centric European identity (Hansen 2002; Hipfl and Gronold 2011) and the use of western-centric constructs as idealised yardsticks of EU norms (Kinnvall 2016; Fitzpatrick and Bergeron 1998; Zielonka 2007). Recent explorations applying postcolonial theory to Europe have focused on the role that race, ethnicity and economic differences play in constructing contemporary identity and policies (Ponzanesi and Blaagaard 2011). They have tended to be grounded in cultural (rather than legal^13^) studies and to focus on domestic (rather than supranational) practices.

Notably, in the context of the CEE region, the postcolonial framework has been applied to analyses of the region’s experiences after the fall of the Iron Curtain in 1989, and of the contemporary persistence of the east/west binary (e.g. Verdery 1996; Stenning and Hörschelmann 2008; Kuus 2004; Taylor and Śliwa 2011). For example, scholars have critiqued the Eastern Enlargement process for its neo-colonial export of governmentality and economic control, and its framing as a civilising mission (e.g. Böröcz and Sarkar 2005; Bohle 2006; Kuus 2006; Behr 2007; Böröcz and Kovacs 2001; Buchowski 2006; Engel-Di Mauro 2006). Embedded within the long-standing western othering of the east, these colonial-like processes have supported accession practices that have incorporated CEE nationals as ‘second-class’ EU citizens (Currie 2009) and a subordinate cultural group (Tutti 2010), and which might be linked to long-term unequal outcomes experienced by CEE movers in EU-15 States (e.g. Currie 2009; Kubal 2012; Engbersen et al. 2017; Myslinska 2019; Myslinska 2019a; Fox et al. 2012; Keskinen and Andreassen 2017). The postcolonial agenda has also been helpful in analysing western exclusionary politics targeting CEE movers (Kinnvall 2016), and CEE nationals’ self-colonising consciousness of inferiority (e.g. Kuus 2004; Samaluk 2016a). My aim is to expand such postcolonial critiques to ECJ discourse.

As postcolonial and other critical scholars have noted, inferiorisation can be based on imparting negative symbolic meaning not only to overt phenotypic differences, but also to ethnicity, cultural practices (Omi and Winant 2015) and immigrant origins (Romero 2008; García 2017; Tehranian 2007; Johnson 2004). Such racialisation^14^ is often further complicated by class (Balibar and Wallerstein 1991; Keskinen and Andreassen 2017; Barker 1981). Groups dominant on the global stage—historically, economically, politically or culturally—construct and re-produce other groups as inferior (Grosfoguel 2004). Implicit and explicit discourses, propagated by individuals and by institutions belonging to or reflecting the dominant group, define inferiorised groups’ identities and affect their outcomes (Rattansi 2005; Kushner 2005). Inferiority gets ascribed to not only groups as a whole but also individuals within them, and is reinforced by essentialising all purported group members as inherently similar (Keskinen and Andreassen 2017).

White skin has not shielded CEE movers from racialisation in western States (e.g. Fox et al. 2012; Garner 2012; Keskinen and Andreassen 2017; van Riemsdijk 2010). For example, in the UK, local majorities have attributed ‘alien values’, primitivisation and criminalisation to CEE movers (Halej 2014, p. 111), based on their perceived lifestyle characteristics^15^ (Moore 2013) and being conflated with undocumented migrants (Halej 2014). Such findings echo how CEE movers have been racialised by the media and in political discourse in EU-15 States, as discussed earlier. Similarly, the ECJ has portrayed some CEE movers—such as the Applicant in the famous *Dano* case (discussed below)—as undesirable ‘welfare tourists’ and ‘migrants’, not belonging to the (western) EU polity, largely due to their lifestyle and economic characteristics. Given such examples of inferiorisation, and because east–west power differentials and western ideology othering the CEE region are at the core of my enquiry, postcolonial theory is particularly relevant to this study.

## Methodology

Discourse analysis looks closely at the role of texts and linguistic units in reflecting and constructing polities, collective practices, social relations and individual identities (Chouliaraki and Fairclough 1999). When this methodology is used to scrutinise the relationship between language and power (including its abuse), it becomes critical discourse analysis (‘CDA’) (van Leeuwen 1993). The connection between language and power is mediated through ideology, that is, shared social beliefs—for example, about class or ethnicity—associated with the dominant group’s identity and position, which re-produce its power, while influencing the actions and beliefs of those who are subjugated (van Dijk 1998). Ideology also encompasses the construction and legitimation of practices that further the dominance of the group in power (Chouliaraki and Fairclough 1999). Both the social elites (such as judges or politicians) and the public disseminate ideology (van Dijk 1998; Wodak 2018). When individuals speak, they express the ideology of the group that dominates knowledge production, and this discourse constitutes them as speakers (Wandel 2001). To unpack how power relations, ideologies and values at play behind complex social phenomena are shaped, CDA looks at how expressions of text legitimise dominance of the speaker—such as through uni-directional communication and justification strategies (van Dijk 1993). Furthermore, it strives to uncover what is implicit or hidden, and what voices, values and interests are emphasised, marginalised or suppressed (id).

CDA has been widely used in research pertaining to the creation of polities and policies, including studies of identity politics and discriminatory rhetoric and practices (van Dijk 1998). Recently, CDA has been increasingly applied to studies of ‘European’ identity, especially as impacted by the fall of the Iron Wall and by ongoing migration debates. Much of such analysis has focused on EU-level politics (e.g. Carta and Morin 2014; Lynggaard 2019; Wodak 2018) and on transformations of western national identities due to political and media discourses (e.g. Mole 2007; Bayley and Williams 2012).

On the other hand, CDA analysis has not been prominent in legal studies, with the exceptions of some anthropological enquiries (e.g. Mertz 1992), studies of regulatory processes (e.g. Black 2002) and investigations of specific, pre-defined legal concepts (e.g. Gales 2009). Although CDA has rarely been applied to analyses of case-law, it is well-suited for revealing value-laden aspects of judicial reasoning, its underlying assumptions, as well as power dynamics between various actors affected by case-law (Kalimo et al. 2018), thus complementing dogmatic and doctrinal studies (Ervo 2016). By prioritising and naturalising certain ideologies underlying legal arguments (Kalimo et al. 2018), jurisprudential discourse conveys specific world views, which in turn construct social reality (Ervo 2016). Notably, US critical race scholars have engaged in analyses which resemble CDA approach, to expose how judicial reasoning in select tort, criminal and contract cases perpetuates race- and class-based stereotypes and inequalities (e.g. Spence 1993–4; Kastely 1994; Zalesne 2013).

Being problem-oriented, CDA is concerned more with goals than with specific processes. Hence, it can encompass any methodological approach and any theoretical framework as long as they offer a critical way to study text relevant to social problems (Wodak 2018). Qualitative content analysis proved to be the most suitable method for this study given my critical theoretical framework, the availability of a large number of relevant documents (discussed below), the complexity of the phenomena that I research and the fact my topic has been under-studied (Webley 2010; Krippendorff 1989). Effective CDA also requires a good theoretical stance on the role of discourse in the reproduction of social dominance and resistance (van Dijk 1995). The postcolonial framework appears well-suited to CDA methodology because it centres on differentiation and subordination, and looks closely at the propagation of social power.

I searched the online EUR-Lex database^16^ for all available ECJ^17^ judgements which included, in text, each of the ten CEE country names or their demonyms (for example, ‘Poland’ or ‘Polish’), without date limitations. This produced 1001 judgements for Poland, 720 for the Czech Republic/Czechoslovakia, 262 for Estonia, 224 for Latvia, 267 for Lithuania, 186 for Slovenia, 254 for Slovakia, 533 for Hungary, 238 for Bulgaria and 233 for Romania. The most common types of results were as follows, in this order: observations submitted by CEE governments regarding cases that did not relate to their nationals’ rights; references for preliminary rulings^18^ from CEE courts in cases between CEE companies and CEE governmental entities; references by CEE courts in cases between CEE nationals and CEE governments or CEE companies; Commission actions against CEE States (for not following EU legislation); and cases regarding inter-state cooperation in criminal proceedings or the enforcement of civil judgements (particularly in matrimonial matters). After skimming all these results, I was able to eliminate types of cases that did not pertain to my research aims. For example, I did not find any value-laden comments about CEE States or their nationals in actions brought by the Commission against CEE States. Arguably, both the Commission and the ECJ tend to be deferential when discussing national governments. Furthermore, such cases—as well as actions brought by CEE States against EU institutions—are not especially politicised. Cases pertaining to CEE companies were also very laconic. I focused my review on judgements in cases where CEE applicants were asserting their rights against western governments before EU-15 courts.^19^ I cite the most data-rich ones below, limited to only four cases due to article length constrains.

My search terms, of course, would have overlooked cases involving CEE nationals where their nationality was not mentioned. That, however, occurs rarely. Given that EU rights get triggered due to being a national of one of the Member States and engaging in the right of free movement, the ECJ routinely mentions parties’ nationalities.

Since implicit in any inferiorisation of the east is a comparison with the west, to be able to better contextualise my data about CEE nationals’ representation, I sought to also provide some illustrative examples of how western movers are approached in ECJ discourse. Thus, I conducted a limited search of ECJ judgements pertaining to EU-15 nationals. Although earlier cases might also be illuminating, I decided to focus on post-2004 cases only—not only for pragmatic reasons (to limit the scope of my data set), but also because the cases pertaining to CEE nationals to which I cite in this paper are all post-2004. Moreover, since the most data-rich cases pertaining to CEE claimants which I had found involved (1) criminal proceedings against them and (2) their assertion of the free movement right (and the associated right to social benefits), I narrowed my searches of EU-15 claimants accordingly.^20^ To test the value of this approach, I began with the three most populous EU-15 States: my search produced 49 (criminal proceedings) and 67 (freedom of movement) results for Germany, 48 and 48 for France, and 8 and 3 for the UK. I then expanded it to the other western States,^21^ locating 33 and 26 results for Austria, 26 and 26 for Belgium, 9 and 29 for Denmark, 15 and 10 for Finland, 11 and 7 for Ireland, 22 and 41 for Luxembourg, 22 and 19 for the Netherlands, and 19 and 17 for Sweden.

For all the judgements that I cite in this paper, I also reviewed corresponding Advocate General (‘AG’) opinions if they were available. If the Court deems that a case raises a new point of law, it requests an AG to prepare independent, single-authored written advice (Parliament 2019), after oral hearing and before judicial deliberations. Although non-binding, AG opinions are valuable for my purposes because they tend to address legal issues more comprehensively than the Court does, often get cited by later AG opinions, and are followed by the Court in the majority of cases (Arrebola et al. 2016). Notably, the language of AG opinions is sometimes reflected verbatim in ECJ judgements. Moreover, AGs tend to be well-respected European legal scholars, legal practitioners and highly ranked public servants, and many later become ECJ judges. Hence, their discourse helps to shape institutional ECJ rhetoric.

## Case Analysis

### Criminal Cases

Although EU competence in the field of criminal law is limited, it includes some harmonisation of substantive and procedural criminal laws (Mitsilegas 2016), Member State law enforcement agencies’ cooperation, and mutual recognition of judgements and orders. For example, the 1990 Convention Implementing the Schengen Agreement (‘CISA’), to which all Schengen area^22^ Member States subscribe, pertains to supporting police and judicial cooperation. Under Article 54, which adopts the *ne bis in idem* principle, a person whose trial has been disposed of in one Member State may not be prosecuted in another for the same acts.

Furthermore, under Council Framework Decision 2002/584 (‘Framework Decision’),^23^ which has been transposed into national laws by all Member States, national judicial authorities may issue a European Arrest Warrant (‘EAW’) to require other Member States to transfer a criminal suspect or sentenced person to the issuing State so that the person can be prosecuted or complete a detention period.^24^ Such persons are provided numerous procedural protections. For example, the executing State must refuse to surrender a requested person who had been acquitted or had already served a sentence for the same act (Article 3). Moreover, the executing authority *may* refuse to surrender a person who has been prosecuted in that State or a third country for the same act, if the prosecutorial authorities in the executing State could not or had decided not to prosecute the person, or if the alleged offence was committed in the executing State (Article 4). Notably, under Article 4(6), the executing State may refuse to surrender a requested person who is ‘staying’ in or is a ‘resident’ of the executing State, if the State undertakes to execute the sentence in accordance with its own law.

#### Criminal Proceedings Against Szymon Kozlowski

In *Criminal Proceedings against Szymon Kozlowski*,^25^ German judicial authority was asked to execute an EAW issued by a Polish court against a Polish national who was serving a custodial sentence in Germany. He argued that Germany should use its discretionary power under Article 4(6) to refuse to execute the EAW because he was either a ‘resident’ of or ‘staying’ in Germany—due to having visited it on numerous occasions and having worked there on various building sites for more than a year before his arrest. He also pointed that he was intending to remain and work there after his release. The German authority disagreed, arguing that his only purpose in being there had been to commit crimes. The German court enquired: (1) whether a person in Mr. Kozlowski’s circumstances could be considered a ‘resident’ of or as having been ‘staying’ in the executing State; and (2) whether Germany’s transposition of the Framework Decision which prevents EAW execution against German nationals opposing surrender but permits such extradition of nationals of other Member States was compatible with EU non-discrimination principle and EU citizenship rights.^26^ In addition to the AG’s opinion and arguments presented by the parties, observations were submitted by nine Member States (including Poland) and by the Commission.

Addressing the first question, the ECJ noted that discretion under Article 4(6) not to execute the EAW was connected to the requested person’s chances of reintegrating into the executing State’s society after the sentence ends. The ECJ concluded that Mr. Kozlowski was not a resident in Germany because Germany was not his actual place of residence. Next, the ECJ instructed that in interpreting ‘staying in’, national authorities must make a holistic factual assessment—based on factors such as the length, conditions and nature of presence, and any family or economic connections—to determine whether ‘a stable period of presence’ had resulted in acquiring connections of a similar degree as those resulting from residence. Given Mr. Kozlowski’s length and nature of stays in Germany, absence of family ties and only weak economic connections, the Court then ruled that he could not be regarded as ‘staying in’ Germany. Thus, discretionary power was not available to Germany to refuse surrendering him pursuant to the EAW. After noting that Article 4(6) was not applicable to Mr. Kozlowski, the Court than declined to address the second question.

##### ECJ Discourse

The ECJ presents itself in this ruling as authoritative and its reasoning as undisputed, as is typical of its judgements (Kalimo et al. 2018). Notably, the Court refers to itself in the third person, as ‘the Court’ (seven times), and states that its interpretation ‘must be’ followed (seven times). Its decision resembles a monologue rather than a deliberation of various viewpoints. For example, the ECJ only acknowledges in passing some Member State observations, without engaging with them, and instead channels them through the Court’s own voice. Notably, observations submitted by the Polish government are not mentioned at all, despite the fact that the issuing government clearly had strong interests in the case. The Court also does not mention the AG’s opinion—perhaps because its approach differs from the Court’s in some significant ways, as described below. What the Court does devote much time to, however, is a detailed description of EU law, which goes beyond statutory provisions relevant to its decision. It also focuses on one guiding value: maintaining uniformity of the Framework Decision’s application across the Member States. Both of these techniques serve to reinforce the importance and authority of EU law.

What is missing in the Court’s substantive discussion is particularly revealing. The ECJ declines to address the second question altogether, even though its determination could have made the first question superfluous (as emphasised by the AG). In fact, the Court does not mention fundamental rights or any rights of requested persons—topics which were discussed at length by both the German government and the AG. By not engaging with arguments about fundamental values or movers’ rights, the Court creates the impression that Mr. Kozlowski is not the type of person for whom EU citizenship and fundamental rights were created.

Furthermore, the Court does not give Mr. Kozlowski a voice. It does not refer to his arguments (other than the fact that he was opposing his extradition). The Court’s discursive approach towards him is also illuminating. Other than in its recitation of the factual background and of the referring court’s questions, the Court refers to Mr. Kozlowski by his name only once. Throughout the rest of its discussion, the Court calls him ‘the requested person’ or ‘the person concerned’, dehumanising him and emphasising his criminal background. It thus becomes easier to other him as inferior (Keskinen and Andreassen 2017). One of the goals of the postcolonial framework has been to disrupt such practices, and to give a voice to those who have been silenced by the dominant knowledge production (e.g. Spivak 1999).

Moreover, the Court includes numerous factual details, only tangentially relevant to the questions at issue, which portray Mr. Kozlowski in a negative light. For example, the Court notes that Mr. Kozlowski was sentenced to imprisonment in Poland for the ‘destruction of another person’s property’ (para 19), and was serving a custodial sentence in Germany of 3 years and 6 months to which he was sentenced ‘by two judgements …. in respect of 61 fraud offences committed in Germany’ (para 20). Ignoring the fact that Mr. Kozlowski had been employed in Germany on construction sites for more than a year, the ECJ infers that ‘his successive periods of presence on German territory were characterised by the commission of several crimes, without any lawful activity’ (para 23). The Court also mentions that he was ‘single and childless,’ and had drawn unemployment benefits in Poland for approximately 1 year (para 25).

All these details paint a picture of someone who is not fit to be an EU citizen, and hence does not deserve discretionary protection from extradition. Mr. Kozlowski is inferiorised as someone whose alien values and lifestyle attributes make him an outsider to the idealised EU polity, not able to integrate there (Omi and Winant 2015). Instead of focusing on traits pertinent to him as a legal actor, the Court re-produces knowledge which is in line with how CEE movers tend to be portrayed by western media and by the western public—as alien, primitive and criminal. Thus, through both institutional and bottom-up knowledge production, persons such as Mr. Kozlowski become racialised as outsiders and deviants (Kushner 2005).

##### AG Opinion

The AG’s Opinion^27^ is much longer than the Court’s, which is not atypical. Since AGs have lesser authority than the Court, they typically rely on other actors’ arguments more. The AG engaged with the German government’s arguments and with other Member States’ observations, including those of the Polish government. Unlike the Court, the AG concluded that Mr. Kozlowski’s intermittent presence, lack of stable resources and commission of crimes in Germany did not preclude a finding of his ‘residence’ or ‘staying in’ there, which should be determined through a holistic factual analysis by the referring court (para 175). The AG also found that Germany’s transposition of Article 4(6) violated non-discrimination rights because it prevented EAW execution against German nationals opposing surrender but permitted such extradition of nationals of other Member States.

The AG’s discussion helps to shed further light on both the gaps and extraneous matters in the ECJ’s discourse. Its substantive and discursive details are particularly revealing. Notably, by addressing at length the second issue in the case, the AG emphasised additional values and interests relevant to the case—including not only requested persons’ rights under the Framework Decision, but also more generally, EU citizens’ fundamental rights to free movement, equality, and re-integration and social rehabilitation following incarceration. In its discussion, the AG drew on the EU Charter of Fundamental Rights and the European Convention of Human Rights, which do not appear to have been discussed by the parties, increasing its appearance of authority as a moral arbiter. Even if the inclusion of such details was driven by the AG’s ultimate legal conclusions rather than by being sympathetic to Mr. Kozlowski, it nevertheless acknowledged Mr. Kozlowski’s EU rights and, more generally, the rights of movers in his position.

In parts, the AG’s discursive approach towards Mr. Kozlowski also appears starkly different than the ECJ’s, even bordering on sympathetic. The AG voices Mr. Kozlowski’s arguments, noting that he had intended to be in Germany to find work, but had fallen into bad company, and that he wished to remain in Germany following his release (para 34). Moreover, throughout the opinion, the AG refers to him by his surname, and often paints background details in a somewhat less negative light than the ECJ does. For example, instead of specifying that Mr. Kozlowski had been convicted of 62 fraud offences, the AG mentions his ‘numerous fraud offences’ (para 31); instead of pointing out that he had received unemployment benefits in Poland for 1 year, the AG notes that he had gotten by with his parents’ assistance and his receipt of ‘limited unemployment benefits’ in Poland (para 30). Unlike the ECJ, the AG also mentions that Mr. Kozlowski had trained as a chef (para 32). Thus, the AG presents a fuller picture of his background and creates an impression of someone who might had experienced some difficulties but nevertheless belongs to the EU polity or at least deserves a chance to be rehabilitated. Of course, the different substantive conclusions reached by the AG and the ECJ might help to explain, at least in part, such discursive differences.

On the other hand, the AG does re-state some negative facts from the referring court’s judgement. For example, the AG notes that Mr. Kozlowski is ‘unmarried and childless’ and ‘has been an alcoholic since 2003’ (para 32). It is not clear whether the AG includes these details unreflexively, simply because the referring court had done so, because the AG himself harbours a negative attitude towards Mr. Kozlowski, or to increase the legitimacy of his opinion by including facts that might go against his legal conclusions. What matters, however, is that such language gets re-produced and normalised, filling institutional discourse with negative symbolic meaning about persons such as Mr. Kozlowski (Rattansi 2005).

#### Criminal Proceedings Against Vladimir Turansky^28^

Austria instituted criminal proceedings against Mr. Turansky, a Slovak national, suspecting him of having carried out a robbery of an Austrian national in Austria. Upon learning that Mr. Turansky was in Slovakia, Austrian authorities stayed their proceedings and requested the Slovak Republic to open proceedings against him. Slovakian authorities opened criminal proceedings into the reported acts, and called Mr. Turansky as a witness. After examination of the merits, the Slovak prosecutor suspended the proceedings, without charging him, and requested that Austrian proceedings also be suspended. Austrian court then referred to the ECJ the question of whether the *ne bis in idem* principle under the CISA precluded prosecution in Austria. The Court ruled that it did not because the decision in Slovakia did not constitute a final disposition. The Commission and seven Member States submitted observations, but the Court chose to proceed without AG’s written opinion.

##### ECJ Discourse

In several respects, the Court’s discourse in this case resembles its legitimating discourse in the *Kozlowski* proceedings. For example, throughout the judgement, the Court refers to itself in the third person and proclaims how its interpretation ‘must’ be followed. Although the Court does mention the arguments of several Member States and of the Commission, it does so only when the Court agrees with them and only in passing, channelling them through the Court’s own voice. The only interest that the Court mentions is the prevention of double prosecution, which is an undisputed principle. Mr. Turansky’s interests are not addressed at all, albeit that might be attributable in part to the fact that he had not submitted observations and did not make an appearance at the hearing.

Notably, the ECJ repeatedly portrays Mr. Turansky—a *suspect* in Austria, against whom proceedings in Slovakia were discontinued on the merits—as condemnable. For example, the Court characterises the alleged crime as ‘a serious robbery’ (para 2) and notes that Mr. Turansky was ‘strongly suspected of serious robbery’ (para 18). These, of course, are subjective interpretations rather than legal concepts. The Court also notes that the alleged crime was committed against an Austrian person ‘at his home’ and that the accomplices had ‘seriously injur[ed]’ him (para 17), adding to the reprehensibility of the alleged crime (and potential culpability in the underlying criminal proceedings). Finally, mentioning that his alleged accomplices ‘are being prosecuted separately’ (para 17) only adds to the impression that the alleged crime did in fact occur. Mr. Turansky is thus implicitly characterised by the Court as a criminal and someone who does not belong to the proper EU polity (Rattansi 2005).

Moreover, the Court does mention several irrelevant negative details regarding not only Mr. Turansky, but also other CEE nationals. For example, the Court notes how Mr. Turansky is suspected of having committed the robbery ‘in the company of two Polish nationals who are being prosecuted separately’ (para 17). Their ethnicity and even the fact that Mr. Turansky might have engaged in a conspiracy are legally irrelevant to the referred questions.^29^ Racialisation of the CEE region operates through ascribing inferior practices not only onto individuals but also to whole groups, through essentialising their purported members as similar and innately subordinate to the idealised yardstick of western norms (Kinnvall 2016; Keskinen and Andreassen 2017).

The case is not accompanied by a published AG opinion.^30^

#### Comparison to How Western Applicants Are Represented

My targeted review of criminal proceedings pertaining to western claimants further illuminates how CEE nationals are inferiorised and their interests overlooked in ECJ discourse. In the cases I had analysed, it became apparent that both the ECJ and AGs tend to refer to western claimants by their surnames, engage with their arguments, and do not infer or imply negative views about them as individuals or about their ethnic groups.

For example, Mr. Kretzinger,^31^ a German national, was convicted twice (in absentia) in Italy for two separate incidents of transporting contraband cigarettes by lorry and not presenting them for customs clearance. He was then sentenced in Germany for evasion of customs duties when the cigarettes had first been smuggled into the EU. The ECJ ruled that, since the two prosecutions pertained to the same act, his sentence in Germany was barred under the *ne bis idem* principle. Other than mentioning that the two sentences had been entered into his criminal record, the Court does not categorise him as a criminal or portray him in a negative light. Instead, throughout its discussion, the Court refers to Mr. Kretzinger by his surname and calls him ‘defendant’ only in the context of the underlying national proceedings. Moreover, the Court engages with his arguments pertaining to each of the three referred questions (paras 41, 47, 57, 59). The discourse adopted by the AG^32^ is very similar.

The Court does not portray western applicants as morally condemnable, regardless of how reprehensible their underlying crimes are. For example, Mr. Mantello^33^ was sentenced in Italy to more than 3.5 years of imprisonment (upheld on appeal) for unlawful possession of cocaine with intent to distribute. After his release, based on wiretap and investigator shadowing evidence, Italian authorities issued an EAW against him^34^ and 76 other persons suspected of a related crime of engaging in international cocaine trafficking and supplying cocaine to a minor. Pursuant to this EAW, Mr. Mantello was arrested at his home in Germany. He objected under the Framework Decision, by arguing that the two actions were based on the ‘same acts’. Despite ruling that the EAW should be executed and despite any moral reprehensibility of the underlying acts, the Court does not portray him in a negative light. In fact, throughout its judgement, the Court notes that Mr. Mantello was merely a suspect (under the EAW)—despite the fact that he had already been convicted in Italy of related crimes. Moreover, the Court consistently refers to Mr. Mantello by his surname, even though he had not submitted observations and was not even present at the hearing. This is starkly different from how the Court had approached Mr. Turansky, the only other applicant discussed in this paper who was not represented and not present at the hearing. The AG^35^ relies on a similar discourse.

Even in cases I had reviewed where EU-15 claimants had already been convicted of the specific crimes directly at issue in their ECJ applications, ECJ discourse gives them a voice and does not condemn them. For example, *Criminal Proceedings against Van Esbroeck*^36^ concerned a Belgian who was sentenced in Norway to 5-years’ imprisonment for importing four types of illegal narcotics. He was then sentenced in Belgium for illegally exporting the same drugs. Throughout their discussions, both the Court and the AG^37^ refer to him by his surname and make no negative inferences about him. Similarly, *Criminal Proceedings against Kraaijenbrink*^38^ involved a Dutch national sentenced in the Netherlands to a suspended 6-month imprisonment term for several offences of receiving and handling proceeds from drug trafficking. Subsequently, she was sentenced in Belgium to 2 years’ imprisonment, for money laundering the proceeds of those drug operations. In their analyses, both the Court and the AG^39^ repeatedly engage with her contentions, refer to her by her surname, do not label her behaviour as criminal, and refrain from making any negative allusions.

Unlike Messrs. Kozlowski and Turansky, EU-15 claimants in these cases are not excluded or denounced as inferior to the norm. They are not treated as second-class EU citizens or uncivilised and alien individuals, or essentialised as such exemplars of their (racialised) ethnic groups. Instead, discourse towards them tends to be neutral and respectful, and the Court voices their concerns. It is true that these applicants are not extolled or applauded by the Court, but that is understandable. Praising such claimants as idealised members of the western norm would have been illogical and would have decreased the Court’s credibility. After all, most are criminal convicts. Moreover, as postcolonial theorists point, racialisation of those perceived as not belonging to the polity is not always complemented by affirmative positive statements about those who belong. Instead, it is simply taken for granted that the latter belong and constitute the norm.

Although the two sets of cases that I analyse are illustrative only and too small for a truly meaningful comparison, they are nevertheless suggestive and indicate some discourse patterns that demand scrutiny. How can such discourse differences in how the Court approaches CEE and EU-15 applicants be explained? Both sets of cases were decided within the same time frame (2006–2010), so within the same socio-political context.^40^ One might wonder whether negative discourse appears correlated with negative substance. After all, court credibility is better maintained when there is no dissonance between discourse and the substantive outcome of its rulings. It is true that the actual rulings produced negative outcomes for both Mr. Kozlowski and Mr. Turansky, whereas three of the four western claimants discussed (Kretzinger, Van Esbroeck and Kraaijenbrink) benefited from the respective ECJ rulings in that the *ne bis in idem* principle was found to bar their later prosecutions. However, even in the one western case with a negative outcome (*Mantello*), the Court did not adopt inferiorising discourse. Moreover, the underlying crimes were less morally reprehensible in CEE than in western applicants’ cases: suspected robbery (in *Turansky*) and property destruction and fraud (in *Kozlowski*); versus drug-related crimes, including a drug ring (in *Mantello*, *Van Esbroeck* and *Kraaijenbrink*), and smuggling contraband goods (in *Kretzinger*). Also, all four western applicants had already been convicted of crimes central or related to their ECJ applications, whereas only one of the two CEE claimants (Mr. Kozlowski) had been. Thus, negative discourse is not clearly correlated with the underlying substance of the cases discussed. Importantly, what both postcolonial theory and CDA emphasise is that both implicit and explicit language is power, by re-producing and naturalising inferiorisation, along with concomitant subordination and unequal outcomes. Thus, it does not matter whether inferiorisation affects or offers additional support for the actual case outcomes or is related to the substance of underlying domestic cases. What matters is that such discourse exists.

### Free Movement Cases

As a cornerstone of the single market, freedom of movement of persons^41^ constitutes a central aspect of the EU integration project. Serving not only as a tangible symbol of integration, but also as a prerequisite for the exercise of most other EU rights, including the right to equality (Johns 2013), the right of mobility carries great social, economic and political importance. Notably, all the cases discussed in this subsection were decided by the ECJ composed as Grand Chamber or Full Court, pointing to the importance of this right to the evolution of EU law.

Directive 2004/38 (the ‘Free Movement Directive’)^42^ provides all EU citizens with the right to reside in other Member States for up to 3 months. For longer stays, economically inactive movers must have comprehensive health insurance and ‘sufficient resources’ so as ‘not to become a burden on the social’ welfare system of the receiving State (Article 7(1)). After 5 years of lawful residence in a host State, movers gain the right of permanent residence (Articles 16–17). From day one of qualifying as a ‘worker’ or a jobseeker in a receiving State, access to that State’s social security benefits,^43^ social and tax advantages^44^ and social assistance (Article 24(2)) follows. After the first 3 months of residence, economically inactive movers are also granted equal access to social assistance, as long as they can demonstrate financial self-sufficiency (Article 24). Although host States are permitted not to grant first-time jobseekers from other States any social assistance (Article 24(2)), the ECJ has mandated that they be given equal access to financial benefits ‘intended to facilitate access to the labour market’.^45^

Notably, the free movement right has been a highly politicised issue in the context of CEE nationals’ mobility. It constituted one of the most contested topics during the Eastern Enlargement process, unpopular among western citizenry and officials (Currie 2016). Allegedly fearing ‘welfare tourism’—although studies indicated that such concerns were not warranted (Dougan 2004)—EU-15 States imposed temporary restrictions on CEE workers’ mobility for up to 7 years after the Eastern Enlargement.^46^ When these transitional limitations were coming to an end, renewed popular and political debates about ‘benefit tourism’ and ‘poverty immigration’ spread across EU-15 States (Galgóczi et al. 2011; Poptcheva 2014). Reflecting such western concerns, the ECJ has been limiting the rights of economically inactive movers, starting with its decisions in *Brey*^47^ and *Dano* (discussed below) (Myslinska 2019). This recent line of cases, consistent with the increasingly restrictive EU-15 policies targeting CEE movers (Eurofound 2014), propagates the notion of EU citizens’ need to ‘earn and deserve’ their right to move and to draw on social benefits (Kramer 2016).

#### Ziolkowski and Szeja v Land Berlin^48^

This joint case pertains to Mr. Ziolkowski and Mrs. Szeja, Polish nationals who had arrived in Germany in the late 1980s and were granted a right of residence on humanitarian grounds. In 2005, Mr. Ziolkowski, Mrs. Szeja, and her two children (born in Germany) applied for permanent residence in Germany. Their applications were refused because they were found unable to support themselves economically. The German court referred the following questions to the ECJ: (1) whether periods of residence in the host State completed in compliance with national law alone may be regarded as periods of legal residence under the Directive; and (2) whether periods of residence completed by nationals of a Member State before its accession to the EU must be considered in calculating the 5-year period for acquiring permanent residence under the Directive. The Court was presented with the parties’ arguments, the AG’s opinion, and observations submitted by three Member States and by the Commission. The Court concluded that the term ‘legal residence’ referred only to periods of residence in compliance with the Directive conditions (that is, being a worker or having sufficient resources). Although the Court answered the second question in the affirmative, it noted that any pre-accession periods of residence also had to be completed in compliance with the Directive’s conditions. Due to this formalistic reading of the Directive’s residency requirements, the Applicants could not prove their right to residence.

##### ECJ Discourse

Similarly to the criminal cases addressed earlier, the Court employs various discourse practices to present itself as authoritative and its decision as logical—by referring to itself in the third person, laying out EU law in great detail (beyond what is relevant to the questions before it), and repeatedly relying on phrases such as ‘it must’, ‘it follows’ and ‘it is apparent’. Moreover, when describing its interpretation of EU law, the Court refers to ‘settled’ case-law and notes that there is ‘no valid basis for contrary interpretation’ (para 32). When discussing question one, the Court mentions select observations submitted by the Commission, the referring court and some Member States, but only parenthetically and only when the Court agrees with them or when the Court’s contrary position is easy to defend, thus increasing its legitimacy. It does not mention the AG’s opinion at all, perhaps in part because the AG had reached different conclusions than the Court.

The Court’s treatment of the Applicants, and of movers more generally, is dismissive. It makes no mention of Mr. Ziolkowski and Mrs. Szeja’s arguments and never refers to them by their names other than when laying out the factual background. Instead, they are called the ‘applicant(s)’ on ten occasions. More generally, the Court mentions the free movement right and the concept of EU citizenship in passing only, which is surprising given their importance to the EU integration project.

Notably, the Court includes many negative details not pertinent to its decision and makes unwarranted negative inferences about the Applicants. For example, the Court points that Mrs. Szeja’s children’s father is ‘a Turkish national who lives separately’ from them (para 19). Characterising her as a single mother of multiple children whose father is not an EU citizen might tap into negative stereotypes of poor migrant mothers who deviate from the norms of acceptable behaviour (Omi and Winant 2015). It also differentiates her whole family from those who firmly belong to the EU polity based on shared culture and ethnicity (Balibar and Wallerstein 1991). Furthermore, the Court notes that the referring court had found both Applicants not able to support themselves financially, and points how the Directive’s intention was to ‘prevent such persons [from] becoming an unreasonable burden on the social assistance system of the host Member State’ (para 40). These details only add to the image of Mr. Ziolkowski and Mrs. Szeja as undeserving of EU law’s protection and of the right to reside in Germany, while tapping into ‘welfare tourism’ concerns targeting CEE movers in western exclusionary politics and popular discourse. Their racialisation is further supported by drawing class-based and economic differences between those who belong and those who do not (Grosfoguel 2004).

##### AG Opinion

Although the AG^49^ acknowledges movers’ interest in integration and points that movers’ ‘material circumstances’ alone do not determine their integration (para 55), he paints a picture of the Applicants as not being part of the EU polity and not the type of movers for whom EU integration was intended. For example, the AG mentions that Mr. Ziolkowski had resided in Germany since the age of 12, had undertaken part of his education there and had a child of German nationality, and that Ms. Szeja had lived in Germany for 20 years and had children born there (para 56). Although such factual details might have been included simply for pragmatic reasons—to support the AG’s conclusion that periods of residence completed on the basis of national law alone should also be taken into account for acquiring residence right under the Directive—they also tap into negative stereotypes of movers who have children in host States and expect host State support, despite having lived there for decades. Furthermore, the AG does refer to numerous negative factual details not clearly relevant to his analysis. For example, although the AG does not mention the ethnicity of Mrs. Szeja’s children’s father, he does note that the father lives separately (para 24). Moreover, the AG includes additional negative background, which is not repeated by the Court—for example, that Mr. Ziolkowski had not completed an apprenticeship which he had started, was unsuccessful in setting up a cleaning business, and was dependent on social security benefits (para 16).

Notably, although the AG does not directly attribute Mr. Ziolkowski’s and Mrs. Szeja’s financial difficulties to them, he does not connect their problems to any institutional impediments they might have faced in Germany. Thus, implicit in the opinion are the Claimants’ own shortcomings in failing to support themselves and their children. This again taps into western exclusionary politics and recalls popular perceptions of CEE movers as unable to integrate in the west—due to their sexuality, poverty and living and employment circumstances (Moore 2013). Any role that the increasing labour precarity in the west or any institutional discrimination in Germany—reinforced by east/west economic, political and cultural power imbalances—might have had in contributing to their struggles is overlooked in this dominant discourse, which reproduces their inferiority and the prevailing racial stratifications (Grosfoguel 2004; Go 2018).

#### Dano v Jobcenter Leipzig^50^

Ms. Dano and her son, Romanian nationals, had been living in Germany since 2010 with her sister, on whom they depended financially. Ms. Dano was 20 when she had her son and was raising him alone. Although she was receiving child benefit and social assistance for her son, the German government denied her a special non-contributory cash benefit^51^ (‘SNCB’) due to her economic inactivity. The German government argued that she had moved to Germany for the sole reason of claiming benefits, which excluded her from access to SNCBs under German law. The ECJ was presented with the following questions: (1) whether the SNCB at issue fell within scope of Regulation 883/2004 mandating equal treatment between movers and host State nationals in the coordination of social security systems; (2) if so, whether Ms. Dano was entitled to it; (3) if not, whether she should be entitled to it under general EU equality provisions. In addition to the parties, five Member States and the Commission submitted observations.

The Court ruled that the SNCB at issue fell within the scope of Regulation 883/2004 and so could be granted only in accordance with the legislation of the claimant’s State of residence. Moreover, the Court stated that, despite a fundamental EU Treaty right of non-discrimination, the Free Movement Directive allows host States not to confer social assistance to movers present in the host State for more than 3 months who are not workers or financially self-sufficient. Finally, the Court concluded that the Directive, read in conjunction with Regulation 883/2004, does not preclude national legislation which prevents movers who lack the right of permanent residence under the Directive from accessing certain SNCBs. Thus, Ms. Dano was not entitled to the benefits in question.

As a result of this judgement, an economically inactive mover’s application for social assistance benefits results in losing the right to reside (due to lacking sufficient resources), without the need for an individual assessment. Moreover, Member States do not have to provide SNCBs to economically inactive movers (even if they are available under secondary EU legislation)—at least not to those who, like Ms. Dano, had never been employed in the receiving State and did not provide sufficient evidence of having searched for work. In effect, poorer movers and those who move without first securing jobs in host States do not enjoy the same rights as movers who are employed or have financial resources. This disadvantages movers from CEE States, who tend to have few financial resources, engage in precarious employment (e.g. Ciupijus 2012; Drinkwater et al. 2009; McDowell et al. 2009; Barnard et al. 2018) and experience difficulties with having their home qualifications recognised in the west (Maas 2013).

##### ECJ Discourse

Throughout this judgement, the Court uses the same discursive techniques as in the judgements discussed above to present itself as authoritative, logical and impartial. Thus, the Court refers to itself in the third person and uses phrases such as ‘it follows’, ‘it is apparent’ and ‘it must be’ throughout its discussion. It also quotes long passages of EU statutory law, beyond the scope of what is at issue. The Court refers to the AG’s opinion only where it is supportive of its own reasoning, and does not engage with it, but instead channels it through its own words. There is also no mention of any of the (western) Member States’ observations, although they were most likely in agreement with the Court’s reasoning. Perhaps the Court wants to maintain the appearance of impartiality by not referring to arguments of the Member States who clearly stand to benefit from its ruling, which protects host States’ purses.

Despite describing fundamental rights under EU Treaties, the Court only engages with one component of these far-reaching rights — the concept of non-discrimination under the Directive, which is much narrower than under the Treaties. This might have been a strategic decision, facilitating the Court’s decision that movers’ fundamental rights are not violated in circumstances such as in this case. Of course, mentioning fundamental rights, not directly needed to reach its conclusion, also serves to provide the Court with a clout of moral authority and helps to obscure the actual negative impact of this decision on poorer movers.

The Court consistently emphasises only one interest—of preventing movers’ becoming an unreasonable financial burden on host States. Being such a burden is contrasted with gaining the right of permanent residence and with integrating in the host State (para 72), implying that movers who are not financially self-sufficient are incapable of integrating and are undeserving of residence rights. Economic status determines belonging and rights, as class-based racialisation is both portrayed as a long-term circumstance and conflated with negative character attributes (Balibar and Wallerstein 1991).

The Court silences Ms. Dano’s voice by not acknowledging her interests, even though she was represented at the hearing, and by making negative inferences about her. Notably, other than in the factual background section, Ms. Dano is mentioned only once, when the Court asserts that ‘[i]t is apparent from the documents before the Court that Ms Dano … is not seeking employment and that she did not enter Germany in order to work’ (para 66). The Court never explains details of this evidence.^52^ Moreover, the Court points that a ‘Member State must … have the possibility … of refusing to grant social benefits to economically inactive Union citizens who exercise their right to freedom of movement solely to obtain another Member State’s social assistance although they do not have sufficient resources to claim a right of residence’ (para 78). What had led the Court to implicitly characterise Ms. Dano as having entered Germany *solely* to obtain social assistance is not clearly substantiated. Even if she had not originally entered Germany specifically to seek work (para 66), that does not automatically mean that she entered it solely to rely on social assistance. The link between these two concepts is never made clear. She could had entered Germany for many other reasons, such as a desire to be with her sister, especially given that she was a young single mother, or a desire to provide better future opportunities for her child. Moreover, mover’s intentions might change after she enters a host State. All such potential explanations are overlooked. Instead, the Court stigmatises her as not belonging to the western norm by imparting a negative meaning to her social practices (Omi and Winant 2015; Keskinen and Andreassen 2017). Postcolonial framework seeks to expose and ultimately to disrupt such inferiorising constructions (Spivak 1999).

In addition, the Court refers to several negative facts not relevant to its conclusions. For example, it mentions how Ms. Dano had been receiving public support for her child ‘whose father’s identity is not known’ (para 38), had attended school for only 3 years, did not obtain any educational certificates or professional training, could not write in German, had never worked and had not provided evidence of having looked for work (para 39). Such details would have been relevant to a proportionality analysis, which was not applicable in this case. Through such discourse, the ECJ creates an impression of someone who does not deserve protections of EU law. Instead, Ms. Dano is portrayed as an embodiment of a ‘welfare tourist’ and a type of mover who would never be able to integrate — or, perhaps, is even too undesirable for the host State to want her to integrate. Thus, the Court relies on implicit and explicit racialising discourse to present her as an inferior outsider, while affecting her material outcome through its ruling (Kushner 2005).

##### AG Opinion

Similarly to the ruling, the AG^53^ emphasises the importance of protecting host States’ purses. The AG refers to fundamental rights even less than the Court does, and instead prioritises each host State’s interests in setting social benefit amounts ‘in light of its own economic and social situation’ (para 48). To support this view, the AG relies heavily on portions of the AG Opinion from *Brey*,^54^ which addressed host States’ prerogative to prevent economically inactive movers from ‘using the welfare system of the host Member State to finance their livelihood’ (para 94). Again, such references tie into the dominant public and political discourses in western States, while re-producing western values and norms (Kinnvall 2016; Zielonka 2007).

Also like the Court, the AG deprives Ms. Dano of her voice and draws negative inferences about her. The AG does not mention any of her arguments and consistently refers to her and her son as ‘the applicants’, without even capitalising this term. The AG’s discourse is permeated with even more negative facts and negative assumptions about Ms. Dano than the judgement is. For example, the AG mentions that she had been ‘convicted for crimes against property and assets’ (fn 5). The AG then acknowledges that such details, which had been noted by the referring court, do not bear on the questions before the Court (id). It begs the question of why the AG restates them. Also like the Court, the AG explicitly attributes negative intentions to Ms. Dano—that she had moved ‘solely in order to benefit’ from the German welfare system (para 118) and ‘without intending to integrate’ in Germany (para 131). The AG draws these inferences without explaining what evidence supports them. This discourse racialises her as a parasitic outsider to the (western) EU polity, in line with western discourse.

As the AG Opinion progresses, it becomes more explicit in its nefarious assumptions and implications, even going beyond what the German government was asserting. Instead of relying on legal arguments, the AG resorts to citing academic work to support his assertion that Member States should be afforded discretion in awarding social benefits to ‘prevent “abuses of host law” in the form of benefit tourism’ (fn 47). Despite acknowledging that the referring court ‘provides no precise information about the existence of such a risk’, the AG points that allowing movers to access social benefits ‘might encourage immigration of Union citizens whose average income is considerably lower’ than that in the host State (para 133). In addition to mentioning ‘immigration’ in the aforementioned statement, the AG also refers to persons in Ms. Dano’s position as ‘migrants’ (para 133, fn 47) — a concept that does not apply to EU citizens, who are by definition ‘movers’. Thus, in line with western exclusionary politics (Kinnvall 2016) and negative media portrayals (Garner 2012), Ms. Dano and potential movers from poorer (such as CEE) States are characterised as welfare spongers and outsiders to the EU polity. The image of her otherness is strengthened by, incorrectly, setting her apart as an immigrant (Balibar and Wallerstein 1991), reminiscent of how some members of the western public conflate CEE movers with undocumented migrants (Halej 2014). Moreover, without relying on any evidence, the AG asserts that it is ‘likely that, in circumstances such as those in the main proceedings, recourse to the social assistance system will not be temporary but will be prolonged indefinitely’ (para 134). By portraying Ms. Dano and movers of similar backgrounds as hopeless and as permanent drains on western States’ purses, this rhetoric conceptualises them as threatening outsiders, not deserving of EU law’s protections or of belonging in EU-15 States. This is also in line with how the CEE region was incorporated into the EU — as a subordinate cultural group (Tutti 2010), populated by ‘second-class’ EU citizenry (Currie 2009).

#### Comparison to How Western Applicants Are Represented

Among the examples I had reviewed, CEE applicants’ racialisation appears more severe in cases pertaining to their mobility than in criminal cases, likely due to how politicised the former issue has been. The scathing nature of discourse inferiorising CEE movers can be even better appreciated when compared to the Court’s much more embracing approach towards EU-15 nationals’ mobility.

For example, *Collins v Secretary of State for Work and Pensions*^55^ concerned an American who, in his early 20s, had spent just over a year in the UK, as a student and doing casual part-time jobs. He had no other ties to any EU State. Relying on his Irish nationality (obtained as an adult), he arrived in the UK 17 years later, with the apparent intention to seek work. After being in the UK for 8 days, he applied for jobseekers’ allowance, which was denied. Despite ruling that Mr. Collins had no right to reside in the UK under EU law (and thus no right to jobseeker’s allowance), the Court begins its analysis of each of the three questions before it with a thorough discussion of his contentions (paras 21, 34, 45). Moreover, the Court consistently refers to him by his surname and does not express any negative views about his behaviour. Furthermore, the Court does not include many additional facts mentioned by the AG (some of which are relevant to the UK test for jobseekers’ allowance), which could have painted a more negative impression of Mr. Collins. Notably, the Court makes it clear that the label ‘benefit tourist’ could not be applied to him, despite the fact that he sought benefits after having been in the UK for only 8 days and without having any links to the UK. The Court repeats the Commission’s observation that, while the UK’s habitual residence test for access to some benefits may be necessary ‘to avoid ‘benefit tourism’ and thus the possibility of abuse by work-seekers who are not genuine, … in the case of Mr Collins the genuine nature of the search for work is not in dispute’ (para 50). The Court does not provide further details of why Mr. Collins could not be a benefit tourist, other than noting that he became employed in the UK ‘shortly after his arrival’ (id).

The AG^56^ also acknowledges Mr. Collins’ arguments and refers to him by his surname throughout his opinion. Notably, despite mentioning facts that shed a somewhat negative light on Mr. Collins (paras 5–7, 23), the AG does not contemplate any negative implications of such facts. For example, Mr. Collins had left the UK 17 years earlier because he had become unemployed and had to claim benefits. He then spent the following decade working short-term and sometimes part-time in various professions (in sales, teaching and as an aid worker), in the USA, central Africa and South Africa. His application for permanent residence in South Africa was refused. He arrived in the UK on a return ticket. It would have been just as easy to characterise someone seeking jobseeker's allowance in Mr. Collins’ circumstances as an aimless benefit tourist, with no intention of integrating in the UK (or anywhere else), and whose only reason for obtaining Irish citizenship was to access EU rights. But neither the AG nor the Court even imagines such a possibility.

In *Trojani v Centre public d’aide sociale de Bruxelles*,^57^ the Court also characterises a western mover in a positive light, despite factual details that convey an image of a mover whose contributions to the host State appear negligible at best. The case concerned a French national who had been briefly self-employed in Belgium in 1972 in the sales sector, and had returned there in 2000 to stay at a campsite and a hostel for about a year. Next, he secured accommodation and began receiving pocket money for doing odd jobs for 30 h per week at a Salvation Army hostel, as part of his ‘personal socio-occupational reintegration programme’ (para 9). Lacking any financial resources, he applied for Belgian subsistence allowance, which was denied. The Court found that, due to his temporary leave to reside in Belgium, he was a ‘worker’, with a right to claim social assistance. The Court’s references to him by his surname and a discussion of his interests throughout the judgement give him a voice and are in line with its ruling. The Court does not mention many facts (included in the AG’s Opinion) that might paint Mr. Trojani in a more negative light, and also does not draw any negative inferences about him.

The AG^58^ similarly refers to Mr. Trojani by his surname and addresses his interests. Notably, the AG does not even allude to the possibility that Mr. Trojani might be a financial drain on Belgium, despite painting a bleaker picture than the Court does—noting that he has ‘no means of subsistence and has been living temporarily’ at Salvation Army (para 2), which had ‘taken [him] in’ because ‘he has no roof over his head and clearly meets the criteria for being given a shelter’, and which had assigned him tasks ‘as a step towards the reinsertion of the person in need into society’ (para 51).

Through neutral, and at times empathetic, discourse that engages with their interests and arguments, both Mr. Collins and Mr. Trojani are approached as members of the EU polity who could not possibly be undeserving benefit tourists. On the other hand, Mr. Ziolowski, Ms. Szeja and Ms. Dano are portrayed as parasitic outsiders who are incapable of integrating, present a drain on western resources, and are not fully entitled to EU rights. They are not only second-class EU citizens, but unwelcomed outsiders to the proper EU polity.

Such differences in discourse cannot be simply attributed to the substantive outcomes of these cases. It is true that the Court’s rulings in both *Ziolowski and Szeja* and *Dano* produced negative repercussions for the Applicants, in line with the negative discourses employed in those judgments. But, despite their respectful discourses towards the Claimants, the actual substantive rulings in both *Trojani* and *Collins* were not unambiguously positive. The Court determined that Mr. Collins was not a worker under EU law. Moreover, according to *Collins*, EU equality protections do not preclude national legislation which makes entitlement to a jobseeker’s allowance conditional on a residence requirement (as long as it meets the proportionality test^59^). Similarly, although Mr. Trojani was deemed a worker, the Court acknowledged host States’ discretion in determining under what circumstances to provide movers with access to benefits.^60^ In addition, the Court confirmed host States’ ability to limit EU citizens’ Treaty rights to free movement and residence as long as any such limits meet the proportionality test. Thus, in both these cases, the Court emphasised host Member States’ interests more than movers’ interests.

One might be tempted to attribute differing discourse observed in these two sets of cases to the dates when they were decided. After all, transitional mobility derogations imposed against CEE States acceding in 2004 were coming to an end in 2011 (when *Ziolkowski and Szeja* was decided), and against Bulgaria and Romania in 2014 (when *Dano* was decided), prompting massive public fears in the west over an influx of CEE movers. Moreover, during this time period, all EU institutions had begun to curb mobility rights and to tolerate western political discourse and policies targeting CEE movers (Myslinska 2019). That being said, however, timing differences alone cannot explain discourse differences illustrated above. At the time *Collins* and *Trojani* were decided, in 2004, the western public had already expressed concerns over CEE mobility (leading to the imposition of post-accession transitional arrangements). In fact, the notion of ‘benefit tourism’ had already become a highly contested issue in the west before the first wave of the Eastern Enlargement occurred in May 2004. Neither the Court nor the AGs in *Collins* and *Trojani*, however, contemplated that this concept could be applied to western movers.

Moreover, even in later cases, ECJ discourse appears respectful towards western movers, regardless of their potential financial drain on host States and regardless of the substance of the underlying rulings. For example, *Geven v Land Nordrhein-Westfalen*^61^ concerned a Dutch national who, after her statutory maternity period had ended in the Netherlands, was working in Germany between 3 and 14 h per week. Her application for child-raising allowance in Germany was refused because she was not a resident there and was found not to be ‘employed’ under German legislation (which required at least 15 h of work per week). Despite ruling that EU law did not preclude such national legislation making frontier workers engaged in minor employment ineligible for social advantages, the Court does not portray her attempt to obtain child-raising allowance in a negative light. Neither does the AG.^62^ Both voice her interests and refer to her by her surname. Similarly, *Bosmann v Bundesagentur für Arbeit - Familienkasse Aachen*^63^ concerned another western frontier worker’s access to benefits. A Belgian national residing in Germany with her two children (who were studying in Germany) had been receiving German child benefits, which were discontinued after she took up employment in the Netherlands because, at that point, only Dutch law was deemed applicable. The Court ruled that it was up to the German court to determine whether she still resided in Germany and hence were eligible for social security there. Both the Court and the AG^64^ refer to her arguments and to her by her surname throughout their discussions, and do not portray her attempt to obtain benefits in a negative light. By the time *Geven* and *Bosmann* were decided (in 2007–2008), transitional mobility derogations imposed against CEE States acceding in 2004 had already come to the end of their first 3-year period, which had led to renewed western public and political apprehensions about movers’ alleged financial drain on host States. Yet no such concerns are even implied in these two decisions.

Notably, *Geven* and *Bosmann* present stark contrast with how ECJ discourse approaches mothers in *Szeja* and *Dano*. Both Mses. Szeja and Dano are condemned - for being single mothers who draw on host State benefits to support their children, fathered by, respectively, a non-EU national and by someone unknown. Moreover, both are portrayed as the types of persons who are likely to become permanent financial drains on the host States, that is, ‘welfare tourists’ who are incapable of integrating and hence do not deserve access to all the benefits that stem from the right of EU citizenship. On the other hand, Mses. Geven and Bosmann are portrayed as proper, respectable mothers who are entitled to host State support, as long as they satisfy all the eligibility requirements. The Court in *Geven* notes irrelevant details that portray the Applicant as a respectable mother, mentioning that ‘[w]hen her son was born … she was living in the Netherlands with her husband, who worked in that Member State’ (para 7). The AG also includes those details. Notably, in *Bosmann*, the Court never uses the phrase ‘single mother’, which might carry negative connotations. Instead, throughout its judgement, the Court refers to her as a mother who has been ‘bringing up’ her children ‘on her own’ (paras 9, 13). The Court also does not refer to her children’s father(s). Although the AG does call her a ‘single mother’ (paras 11, 32), he does emphasise that national legislation treating a ‘single mother’ less favourably than ‘a mother forming part of a couple’ (para 32) would not be permissible. Thus, unlike Mses. Szeja and Dano, Ms. Bosmann deserves equal access to social benefits to help support her children. Despite being a single mother, she is not inferiorised but instead, she belongs to the EU polity.

## Discussion and Concluding Remarks

The cases discussed in this essay illustrate not only how the ECJ adopts various discursive strategies to maintain its authority,^65^ but also how both the Court’s and the AGs’^66^ discourses tend to explicitly acknowledge and to prioritise values of western Member States and western movers, while overlooking and silencing interests of CEE nationals. This one-sided approach is further reinforced by referring to gratuitous negative factual details and by relying on negative assumptions about CEE applicants. At times, this negative discourse is used by the Court to suggest the substantive outcome of the underlying cases, despite the fact that the ECJ’s role in preliminary rulings is simply to interpret EU law instead of deciding the dispute. Existing scholarship has shown that ECJ judgements tend to defer to the dominant^67^ Member States^68^ (Carrubba and Gabel 2015; Larsson and Naurin 2016) and to take into account western public fears over ‘welfare tourism’ (Blauberger et al. 2018; Kramer 2016). My analysis suggests, moreover, that there are instances where ECJ jurisprudence might not only take into account, but also propagate such antagonistic statements. Furthermore, I have found that the Court inferiorises CEE nationals even in contexts which do not directly pertain to their mobility, such as in criminal^69^ proceedings.

The judgements explored in this paper create and entrench an image of CEE nationals as not quite deserving of belonging to the EU, and as socially and economically inferior to westerners. CEE movers are portrayed as having little knowledge, limited education and insufficient financial resources, and as lacking the ability to conform to proper middle-class behavioural practices. Both explicit details and discursive strategies used by the Court invite the reader to associate CEE movers with illegality, poverty, poor judgement and defect. Such discourse also encourages the reader to rely on an intersection of negative ethnicity- and class-based stereotypes,^70^ creating complex identities that shape access to privilege and intersect with experiences of discrimination and inequalities (Delgado 2011). These stereotypes are in line with today’s nuanced expressions of racisms, based not solely on phenotypical differences, but also on culture, ethnicity and class (Garner 2006; Pruitt 2015). They reify problematic assumptions about ethnicity, class and migration status, overlooking the fact that rates of both criminality (e.g. Wadsworth et al. 2016) and reliance on public welfare (e.g. Dustmann and Frattini 2014; Barslund and Busse 2014) are lower among CEE movers than among western States’ native populations.

Notably, in all the above cases, both the ECJ and the AGs disregard east/west socio-economic and political power differentials and the precarity experienced by many CEE workers in the west, further exacerbated by recently increasing austerity measures and labour flexibilisation across western Europe. They also appear ignorant of well-documented studies indicating CEE movers’ experiences of inequalities and racialisation in the west. By failing to acknowledge such barriers to CEE nationals’ mobility, ECJ discourse leaves the reader with a vague sense of CEE movers’ own incompetence and inability to be deserving EU citizens. It also helps to entrench the status quo, as we are prompted to overlook any connection between intra-EU economic inequalities, anti-CEE antagonism, institutional barriers and exploitative practices in the west, and these applicants’ experiences. Judicial ability to define problems and solutions and the value-laden elements of its discourse not only affect power dynamics between various actors, but also naturalise certain ideologies underlying legal arguments and present a certain world view, thereby constructing a social reality and entrenching stereotypes.

It might at first seem particularly striking that such inferiorising discourse has been propagated in judicial and AG opinions discussed in this article, all of which were written after 2004. Since then, the Court has included judges^71^ and AGs from CEE States,^72^ which likely serves to inhibit at least some expressions of anti-CEE sentiment. Moreover, since ECJ rulings tend not to restate prior judgements’ wordings as much as in the common law tradition, one would expect that the inclusion of CEE members would help to make ECJ discourse less west-centric over time.^73^ However, since the Court speaks with one voice, it is difficult for any less western-centric voices to become prominent, especially given that CEE judges comprise a numerical minority (10 out of 27, currently). Judges of CEE origin constituted the majority of the bench in only one decision discussed in this paper (in *Kretzinger*). Not a single CEE judge was on the panels in *Turansky*, *Collins* or *Trojani*. Currently, 4 out of 11 AGs have CEE origin.^74^ Notably, in the cases discussed here, only one AG was from a CEE State (in *Bosmann*). Moreover, pro-CEE voices might lack prominence in post-04 ECJ discourse due to self-colonisation. As postcolonial theorists have revealed, local elites from inferiorised countries often assimilate the dominant group’s rhetoric in order to conform and improve their status. Behr (2012) has observed, for example, how the success of the EU’s eastern ‘colonialism’ was facilitated by the local elites’ embrace of western discourse (see also Szczerbiak 2002).^75^

Importantly, instead of looking at the identities of individual speakers, the focus of CDA analysis is on how power gets re-produced through deeply entrenched *institutional* discourse. The dominant collective discourse ‘speaks through’ individuals, both those who are embedded within dominant institutions and the public that propagates discourse in a bottom-up manner (Wodak 2018). Similarly, postcolonial theory focuses on language as an expression of the dominant group’s institutionalised power. This institutional perspective is particularly applicable to an analysis of the ECJ’s discourse since the Court’s decisions are not attributed to specific judges.^76^ Although AGs do issue individual opinions, their identity is also never emphasised by the Court. If the Court actually does mention their opinions in its judgements, it refers to them as ‘Advocate General’. Thus, both are best approached as speaking with institutional, rather than individual, voices.

As an institution, the EU was explicitly founded on the promise of equality and inclusion, to promote ‘humanitarian and progressive values’ (Commission 2014, p. 5), including ‘human dignity, freedom, [and] equality’, which form the EU’s ‘spiritual and moral heritage’ (CFR,^77^ Preamble). That same promise underlaid the Eastern Enlargement, framed as a historic task to ‘transcend … former divisions and … forge a common destiny’ (Constitutional Treaty,^78^ Preamble). The European Parliament has commended the ‘active role played by the European Union in the world as a defender of human rights’ (Parliament 2009: ¶ 3). Moreover, according to the ECJ, ‘[f]undamental rights [are] enshrined in the general principles of Community law and protected by the Court’.^79^ Aside from questions about whether such promises have been reflected in actual EU policies, which has been addressed elsewhere (e.g. Myslinska 2019; Currie 2007), how can examples of ECJ discourse discussed in this article be reconciled with such grand visions?

Postcolonial theory might offer some guidance. Existing scholarship has applied postcolonial theory to explore the lasting effects on identity and on outcomes of post-war east/west relations. The Eastern Enlargement and the broader ‘Europeanisation’ processes have been shown to prioritise western economic and political interests and to situate western culture as the ideal (Böröcz and Kovacs 2001). Moreover, scholars have applied the postcolonial framework to explore how CEE workers in the west are approached by employers and by the wider public (e.g. Samaluk 2016; Loftsdottír and Jensen 2012), and how self-colonisation impacts their perceptions of themselves (e.g. Samaluk 2016a). This paper expands the application of postcolonial theory to contemporary east–west relations within the EU by illustrating how both CEE individuals and CEE groups are implicitly and explicitly racialised in ECJ discourse based on their cultures, ethnicities, class status and ‘immigrant’ background. Eastern voices, histories and interests are ignored, helping to continue the effects of ethnic differences in economic, political and knowledge-production stratifications. The historical and contemporary political and economic processes that have supported the creation of these stratifications get overlooked. In the process, such discourse has the power to not only constitute identities, but also affect outcomes through delineating access to privilege and facilitating experience of discrimination.

Of course, application of postcolonial theory to the EU is complicated. I am aware that my analysis might problematise the framework by divorcing it from its original context and stripping it to its bare components. Notably, the CEE region never comprised actual colonies of the west, and west’s exploitation of the eastern peripheries of Europe pales in comparison to the great colonial powers’ pillaging areas outside of Europe. Both CEE and western Member States are comprised of majority Caucasian populations and imagine themselves as ‘white’, even if some do have significant numbers of BAME individuals. CEE Member States are formally afforded a proportionate say in EU institutions, and all EU nationals are formally equally protected by EU laws. Of course, formal equality does not ensure equality in practice (Hepple 2014); nor does it preclude negative rhetoric. Despite all these differences from the contexts which first inspired postcolonial theory, application of the postcolonial framework to my project has proven fruitful for helping to advance the framework’s goals — by exploring institutional racialisation of CEE nationals, and revealing (and hopefully helping to disrupt) the persistent east/west binary within contemporary Europe. According to Said’s (1983) travelling theory concept, theories and ideas can be re-invigorated by new contexts in which they are applied. Showing postcolonial framework’s usefulness in settings that are novel to its application is a testament to its great malleability, credibility and continuing value. The framework can still generate new insights into how law and society operate in the context of racism and subordination, and can provide a basis from which to examine today’s nuanced forms of racial stratifications and splintering axes of disadvantage.

My findings also have implications for the EU as an institution. For one, the apparent one-sidedness of ECJ jurisprudence and failure to consider counterarguments and diverse voices is problematic for its legitimacy. Better engagement with applicants’ voices is particularly crucial in cases that implicate their fundamental rights, such as those discussed in this paper, all of which pertain to criminal and mobility protections. In addition, discourse that is not in line with the substantive ruling of a case offers especially poor guidance for national courts and for private individuals, while further reducing the ECJ’s credibility and authority.

More importantly, this article sheds further light on EU power dynamics. While I acknowledge that ‘Europe is a complicated maze with many fault lines, not one single fault line, between the East and the West’^80^ (Zielonka 2019) and we should resist any attempts to essentialise the CEE region as one homogeneous block, there are nevertheless key differences between the east and the west which might fuel and be reinforced by inferiorising institutional EU discourse. For one, although economic gaps between the pre- and post-04 Member States have been decreasing over time, CEE States collectively continue to significantly lag behind the others in terms of their GDPs and wages.^81^ CEE States have had a history of emigration to the west, and its nationals have faced a history of subjugation to anti-Slavic stereotypes. There is also a fundamental difference in how western and eastern Member States perceive the concept of Europe — the former as a way to increase their global power, the latter increasingly as a threat to their identity and very existence (Gallon 2019). Moreover, even if the east/west divide is more psychological than substantive, it does affect the relationship between pre- and post-04 Member States and has an impact on policy decisions (Lehne 2019). Western States have on occasion made CEE States feel like outsiders. For example, in a 2013 letter to the European Council for Justice and Home Affairs, representatives of Austria, Germany, the Netherlands and the UK had complained about the alleged financial abuse of western ‘benefit magnet’ Member States by CEE ‘immigrants’. During the Versailles summit in 2017, France, Germany, Italy and Spain endorsed a two-speed Europe, prompting irritation in the CEE region (Möller and Pardijs 2017). Furthermore, after Brexit, the two London-based EU agencies will be relocated to Paris and Amsterdam, despite the fact that most of the CEE States had requested to host them^82^ and some do not host any EU agencies yet.^83^

Notably, analyses of Member States’ influence on EU policy-making have revealed that the CEE States tend to wield less power than their western counterparts. It might come as no surprise that, 5 years after the Eastern Enlargement, Germany was overrepresented in the European Parliament’s governing bodies, followed by France, Italy and the UK, in line with their voting weight for Council decisions under the qualified majority voting rules (Euractiv 2009). What is more troublig, however, is that more recent studies have continued to show similar patterns. For example, Germany, France, Italy and the UK have continued to hold greatest voting weight for Council decisions after 2014 changes to the qualified majority voting rules.^84^ According to a survey conducted in 2018, experts perceive Germany and France as by far the most influential Member States, followed by the Netherlands, the UK and Italy (Janning 2018). As Lehne (2019) notes, Germany and France have always been at the heart of European integration. A study of MEPs’ influence over Parliament decisions during the 2017 autumn term revealed that the ten most influential MEPs were, in this order, Italian, German, Italian, Belgian, Italian, British, Spanish, Spanish, Polish and Spanish. Taking State populations into account (since it determines the number of MEPs per State), Germany and Italy exert the biggest level of influence in the Parliament (VoteWatch Europe 2017). Looking at the influence of individual MEPs collectively by country, Germany and Italy are again the most influential (id). When it comes to Member State influence over the Council, a study of votes between 2010 and 2017 revealed that France, Italy and then Germany had the greatest impact (Frantescu 2017). Inferiorisation of the CEE region and its nationals in EU discourse makes such findings more poignant.

It is uncertain how recent events might reshape these dynamics. Due to Brexit, French and German influence is predicted to increase further (Lehne 2019), and new coalitions and new fractures might arise. Notably, in the case of Poland—which has wielded more influence within the EU than other CEE States^85^—its ally in the EU will depart and its role of bridging differences between the Big Three^86^ (Parkes 2013) will end. On the other hand, Poland will become the fifth largest State, with a greater share of MEPs in the Parliament. In addition to Brexit, the lack of a harmonised response to Covid-19 crisis and its predicted economic repercussions are likely to further destabilise the EU integration project. Hence, efforts to better understand and to reduce fractures within the EU have become more urgent than ever — based on a better understanding of multiple perspectives, more inclusive policy initiatives and more balanced discourse.

Although this study is based on an analysis of too few cases to draw firm conclusions about prevalence of specific discourses in ECJ jurisprudence, it permits us to observe the prioritisation of specific voices and values, and the marginalisation of others. That being said, I do not dismiss the possibility that multiple and even antagonistic discourses might exist in ECJ and AG opinions. Moreover, texts are open to multiple readings, and this creates space for contestation and change. What is clear, however, is that more academic attention should be devoted to studying discourse produced by ECJ judges and by AGs. Both are in a positions of power to produce dominant discourse as they interpret and legitimise existing legal, political, economic and social arrangements which make such discourse possible in the first place. As this study shows, there are indications that both propagate discourse which others the CEE region and its nationals. The promise of the EU project dictates that we try to make discourse (and policies) more inclusive of contemporary demographic changes and transnational contexts, and more responsive to contemporary varieties of otherings and racisms — imperative today more than ever. The first step is to make racialising discourse visible and to begin de-normalising it. Both researchers and policymakers should endow CEE nationals with a greater voice and should also explore their agency, an essential part of the postcolonial project. Only then will it become possible to imagine—and ultimately, effectuate—a Europe which comes closer to its lofty goals.

## Footnotes

1 CEE Member States include the Czech Republic, Estonia, Hungary, Latvia, Lithuania, Poland, Slovenia and Slovakia, which acceded (along with Cyprus and Malta) to the EU in 2004, and Bulgaria and Romania, which acceded in 2007.

2 Europe Agreement establishing an association between the European Communities and their Member States, on the one part, and the Republic of Poland, on the other part, dated 16 December 1991. Other CEE States entered into similar agreements.

3 Pre-Eastern Enlargement Member States: Austria, Belgium, Denmark, Finland, France, Germany, Greece, Ireland, Italy, Luxembourg, the Netherlands, Portugal, Spain, Sweden and the United Kingdom.

4 Many studies have documented CEE movers’ unequal outcomes (particularly in housing and employment) and experiences of blatant racism and micro-aggression in EU-15 States (e.g. Groenendijk et al. 2013; Kofman et al. 2009), including in the UK, long before the spike in public antagonism associated with the Brexit referendum (e.g. Ciupijus 2012; Drinkwater et al. 2009; Fox 2012; Rzepnikowska 2019; Sime et al. 2017; Johns 2013).

5 Moreover, Kukovec (2015) has shown how EU competition rules and case-law have ignored the concerns of workers from CEE and other ‘peripheral’ Member States (i.e. Portugal, Greece and Cyprus).

6 E.g. Case C-67/14, Jobcenter Berlin Neukölln v Alimanovic; Case C-333/13, Dano v Jobcenter Leipzig; Case C-308/14, Commission v United Kingdom.

7 It was not until 1996 that a major western history of Europe systematically included CEE cultures and nations (Davies 1996).

8 Despite the fact that the EU is ‘primarily a textual enterprise’ and a ‘print community’ (Williams 2003, p. 666), EU discourse generally remains under-studied (Shore 2000), in part because the EU is assumed to be a pragmatic construct (Della Sala 2013).

9 The ECJ’s role should not be underestimated, of course. The Court is the final interpreter of EU legislation. Moreover, it is expected to facilitate European integration by protecting fundamental rights and providing solutions to problems that EU political institutions are unable to tackle collectively (Lenaerts 2013).

10 That is, judges’ expressions of opinion and contextual details not essential to their rulings.

11 The Court’s composition varies between a Full Court of all judges (currently 27), a Grand Chamber of 15 (and formerly, 11 or 13) judges and Chambers of 5 or 3 judges. Only the judges who form the bench in a particular case address it. The Court sits as Grand Chamber if a case raises particularly complex or important issues (or at the request of the Member State or EU institution that is a party), and as Full Court only in exceptionally important cases.

12 Although I look at the CEE region collectively, each CEE State’s unique history, migration patterns, stereotypes and identity affect how its nationals experience mobility and belonging within the EU. For example, Poles have been especially subjected to the superficially positive stereotype about their strong work ethic (MacKenzie and Forde 2009), while their large numbers in western States have made them the face of CEE mobility. Moreover, CEE countries do not all share the same economic, security and political aims (Zielonka 2019). That being said, western rhetoric has tended to inferiorise the whole region (e.g. Cook et al. 2011; Fox et al. 2015; Kofman et al. 2009). All CEE States experienced similar power imbalances with the west during their accession processes, and all are treated alike under formal EU laws. And, within the broad EU backdrop, all CEE nationals have been relatively poor in comparison to EU-15 nationals, as indicated, for example, by their GDP per capita (e.g. Eurostat 2004, 2018), and the CEE block’s average minimum wages (Dascalu 2017).

13 Some European legal scholars have noted the utility of postcolonial studies to legal analyses of racism (e.g. Möschel 2014; Tuitt 2004; Fitzpatrick 1987). Their work, however, does not address intra-EU mobility.

14 ‘Racialisation’ occurs whenever the ‘category of “race” is … evoked in discursive and institutional practices to interpret, order, and indeed structure social relations’ (Fox et al. 2012).

15 Such as their clothing, hairstyles, food and alcohol consumption, gender relations, sexuality, employment, perceived poverty, living circumstances and housing.

16 *See* http://eur-lex.europa.eu/. Managed by the EU Publications Office, this official database includes all EU laws, preparatory documents, legislative histories and Parliamentary questions, dating back to 1951.

17 I did not review decisions of the General Court, given that its most important work is in the field of competition, intellectual property and external trade law. I also did not look at cases before the Civil Service Tribunal, which hears disputes involving employees of EU institutions.

18 When making a reference for a preliminary ruling, national courts refer to the ECJ questions about interpretation of EU law or validity of EU action that had arisen in disputes brought before them. It is then for the national court to dispose of the case in accordance with the ECJ’s decision, which becomes binding on all Member States.

19 I also searched for all ECJ judgements that contained ‘East’, ‘Balkan’ or ‘Baltic’ in text, which produced 360 results. Moreover, searching for A-8 (the term frequently applied to the eight CEE States acceding in 2004) or A-2 (referring to Bulgaria and Romania) in text produced 196 cases, and for EU-8 or EU-2 produced 30 judgements. All of these results were either not relevant or not data rich.

20 Moreover, I only searched for demonyms rather than country names because my search of CEE claimants had revealed that using this approach was more fruitful.

21 I did not look at Greece, Italy, Portugal or Spain because Southern Europeans have faced their own history of negative stereotyping and racialisation by north-western Europeans (Dyer 1997; Ignatiev 2008). Any such racialisation of Southern Europeans presents a separate, albeit likely overlapping, research question.

22 All EU Member States other than Ireland, Bulgaria, Romania, Cyprus and Croatia. Four non-EU countries (Iceland, Liechtenstein, Norway and Switzerland) also belong to the Schengen area.

23 Council Framework Decision of 13 June 2002 on the European arrest warrant and the surrender procedures between Member States, OJ L 190, 18.7.2002.

24 When an offence is punishable by detention for a maximum period of at least 1 year or, in conviction cases, where the remaining term of imprisonment is 4 months or longer (Article 2(1)).

25 Case C-66/08, Judgment (Grand Chamber) of 17 July 2008.

26 This was only the second time that the ECJ was asked to rule on the Framework Decision. *See* Case C-303/05, Advocaten voor de Wereld VZW v Leden van de Ministerraad, Judgment (Grand Chamber) of 3 May 2007.

27 Case C-66/08, Opinion of Mr. Advocate General Bot, 28 April 2008.

28 Case C-491/07, Judgment (Sixth Chamber) of 22 Dec 2008.

29 Albeit any evidence of a criminal conspiracy would be relevant to the underlying criminal proceedings in Austria.

30 AG Bot was present at the hearing.

31 Case C-288/05, Criminal Proceedings against Jürgen Kretzinger, Judgment (Second Chamber) of 18 July 2007.

32 Opinion of Advocate General Sharpston, 5 Dec 2006.

33 Case C-261/09, Gaetano Mantello, Judgment (Grand Chamber) of 16 Nov 2010.

34 Mr. Mantello resides in Germany. His nationality is not listed.

35 Opinion of Mr. Advocate General Bot, 7 Sept 2010.

36 Case C-436/04, Criminal proceedings against Leopold Henri Van Esbroeck, Judgment (Second Chamber) of 9 March 2006.

37 Opinion of Mr. Advocate General Ruiz-Jarabo Colomer, 20 Oct 2005.

38 Case C-367/05, Criminal proceedings against Norma Kraaijenbrink, Judgment (Second Chamber) of 18 July 2007.

39 Opinion of Advocate General Sharpston, 5 Dec 2006.

40 At the time, EU institutions were expanding access to free movement of EU-15 nationals, while CEE workers were being subjected to direct and/or indirect transitional mobility restrictions in the majority of EU-15 States (Myslinska 2019a). *See also* n 46 infra.

41 Along with the freedom of movement of goods, capital and services.

42 Directive 2004/38 of 29 April 2004 on the right of citizens of the Union and their family members to move and reside freely within the territory of the Member States.

43 Regulation 883/2004 of 29 April 2004 on the Coordination of Social Security Systems.

44 Regulation 492/2011 of 5 April 2011 on freedom of movement for workers within the Union.

45 Cases C-22/08 and C-23/08, Vatsouras and Koupatantze v Arbeitsgemeinschaft Nürnberg.

46 During 2006–2009, mobility restrictions were imposed by nine EU-15 States on CEE countries that had acceded in 2004: Austria, Belgium, Denmark, France, Germany, Ireland, Luxembourg, the Netherlands and the UK. During the final phase of transitional derogations, in 2010–2011, Austria, Germany, Ireland and the UK continued to maintain restrictions. Moreover, during 2007–2009, Bulgarians and Romanians were subjected to restrictions in thirteen EU-15 States (all but Finland and Sweden), reduced to ten during 2009–2012.

47 Case C-140/12, Pensionsversicherungsanstalt v Peter Brey, Judgment (Third Chamber) of 19 Sept 2013.

48 Joined Cases C-424/10 and 425/10, Judgment (Grand Chamber) of 21 Dec 2011.

49 Opinion of Advocate General Bot, 14 Sept 2011.

50 Case C-333/13, Judgment (Grand Chamber) of 11 Nov 2014.

51 Subsistence benefit, social allowance and housing and heating costs contribution.

52 All we are told is that Ms. Dano had not provided evidence of having looked for work in Germany (para 39).

53 Opinion of Mr. Advocate General Wathelet, 20 May 2014.

54 Opinion of Advocate General Wahl, 29 May 2013. The *Brey* holding suggested that an economically inactive mover’s entitlement to a means-tested SNCB could be an indication of not having sufficient resources. *See* Case C-140/12, Pensionsversicherungsanstalt v Peter Brey.

55 Case C-138/02, Collins v Secretary of State for Work and Pensions, Judgment (Full Court) of 23 March 2004.

56 Opinion of Mr. Advocate General Ruiz-Jarabo Colomer, 10 July 2003.

57 Case C-456/02, Trojani v Centre public d’aide sociale de Bruxelles (CPAS), Judgment (Grand Chamber) of 7 Sept 2004.

58 Opinion of Mr. Advocate General Geelhoed, 19 Feb 2004.

59 The requirement must be justified on the basis of objective considerations that are independent of the nationality of the persons concerned and proportionate to the national policy’s legitimate aim (to protect State purse).

60 The ECJ found that Mr. Trojani would be entitled to benefits if the referring court determined that his employment activity was real and genuine.

61 Case C-213/05, Geven v Land Nordrhein-Westfalen, Judgment (Grand Chamber) of 18 July 2007.

62 Opinion of Mr. Advocate General Geelhoed, 28 Sept 2006.

63 Case C-352/06, Bosmann v Bundesagentur für Arbeit - Familienkasse Aachen, Judgment (Grand Chamber) of 20 May 2008.

64 Opinion of Mr. Advocate General Mazák, 29 Nov 2007.

65 Confirming the analysis of Kalimo et al. (2018) of ECJ copyright jurisprudence.

66 Some AG opinions appear less negative, while others more negative than corresponding Court judgements in how they describe CEE nationals. Although I analysed here too few cases to draw firm conclusions, this might be attributable to the fact that AG opinions are single-authored and therefore are more likely to reflect individual AGs’ subjective views. Notably, of all the AG opinions discussed in this paper, only one was penned by an AG of CEE background—AG Mazak (from Slovakia) in *Bosmann.*

67 As discussed below, it has been the larger western Member States that have wielded greatest influence over EU policies.

68 And also defer to EU legislators (Lenaerts 2013).

69 Of course, CEE nationals’ alleged inherent criminal propensity has been a part of western anti-CEE exclusionary discourse.

70 And gender-based stereotypes, although that is beyond the scope of my paper.

71 Each Member State appoints one ECJ judge. *See* https://europa.eu/european-union/about-eu/institutions-bodies/court-justice_en.

72 *See* http://www.statewatch.org/news/2019/oct/ep-briuefing-a-g-cjeu.pdf.

73 ECJ judges and AGs are appointed for renewable 6-year terms. Thus, they remain connected to their countries of origin and likely predisposed to reflect interests of their home countries and their nationals.

74 As of 1 May 2020, the Court includes one AG from each of the following Member States: Bulgaria, the Czech Republic, Denmark, Estonia, France, Germany, Ireland, Italy, Poland, Spain and the UK. *See* https://curia.europa.eu/jcms/jcms/Jo2_7026/en/.

75 Of course, the recent pushback by some CEE governments, especially Hungarian and Polish, against some EU policies might be indicative of the gradual evolution of their consciousness away from western norms.

76 Although panel members are listed on each judgement.

77 Charter of Fundamental Rights of the European Union, OJ C 303, 14.12.2007.

78 Treaty Establishing a Constitution for Europe, signed on 29 Oct 2004, OJ 2004/C 310, 16.12.2004 (unratified).

79 Case 29–69, Erich Stauder v City of Ulm – Sozialamt, Judgment of 12 Nov 1969.

80 Some scholars point that CEE States do not have as many political, economic, cultural and policy commonalities with each other as popularly assumed and share more attributes with western States than is commonly acknowledged (Zielonka 2019). Moreover, the east–west divide might be further exaggerated by the media, populist politicians (id) and academics (Lehne 2019).

81 *See supra* note 12.

82 *See* https://www.consilium.europa.eu/en/policies/relocation-london-agencies-brexit/.

83 *See* https://europa.eu/european-union/about-eu/agencies_en.

84 *See* https://www.europarl.europa.eu/RegData/etudes/ATAG/2014/545697/EPRS_ATA%282014%29545697 _REV1_EN.pdf.

85 Poland often closely follows the top influencers. However, it is the only CEE country to do so, and largely due to its population size (38 million). Moreover, it is not clear how receptive western States have been to Poland’s concerns. For example, Copsey and Pomorska’s (2010) analysis of the EU’s policy towards its eastern neighbours—an issue of key strategic importance to Poland—indicated that Poland’s influence on EU policies has been low. Moreover, Euroskeptical policies of its current government have weakened Poland’s ability to form coalitions with western delegations and have decreased its potential clout.

86 France, Germany and the UK.
